# Editorial: Intersection between the biological and digital: synthetic biological intelligence and organoid intelligence

**DOI:** 10.3389/fncel.2024.1542629

**Published:** 2025-01-08

**Authors:** Michael Taynnan Barros, Brett J. Kagan, Thomas Hartung, Lena Smirnova

**Affiliations:** ^1^Computer Science and Electronic Engineering, University of Essex, Colchester, United Kingdom; ^2^Cortical Labs, Melbourne, VIC, Australia; ^3^Bloomberg School of Public Health, Johns Hopkins University, Baltimore, MD, United States; ^4^Biology, University of Konstanz, Konstanz, Germany

**Keywords:** embodied intelligence, artificial intelligence, organoid intelligence, bioengineering, neuroscience, learning, memory, cell culture

The convergence of biology and digital technology is redefining our understanding of intelligence, innovation, and humanity's future. Synthetic Biological Intelligence (SBI) and Organoid Intelligence (OI) are leading this transformation by merging living biological systems with computational frameworks, creating groundbreaking opportunities in medicine, research, and biocomputing. The Research Topic “*Intersection Between the Biological and Digital: Synthetic Biological Intelligence and Organoid Intelligence*” reflects the interdisciplinary nature and disruptive potential of this field. With already nearly 9,000 downloads and 86,000 views at the time of closing the Research Topic, it is evident that this subject resonates deeply with researchers and innovators across the globe. The five articles and contributions from 25 authors provide a comprehensive exploration of this rapidly evolving field, spanning experimental methods, engineering solutions, and ethical considerations.

## The promise of synthetic biological intelligence

At its core, synthetic biological intelligence seeks to enhance the functionality of biological systems by integrating artificial intelligence (AI) technologies. Organoid Intelligence (OI) (Smirnova et al., 2023a), a subset of SBI, demonstrates the potential to revolutionize biomedical research by leveraging organoids—miniature, lab-grown versions of human organs derived from stem cells—as computational models. These models offer unparalleled insights into human biology and disease mechanisms. This Research Topic is part of the attempts of establishing a community to realize this promise (Morales Pantoja et al., 2023; Hartung et al., 2023).

The ability to use organoids as personalized disease models is a significant advancement (Smirnova et al., 2023b). They provide a platform to test drug efficacy and toxicity in patient-specific contexts, moving us closer to truly individualized medicine. Moreover, the potential to model rare diseases and genetic disorders, which often lack effective animal or in-vitro analogs, underscores the societal and medical value of this research.

## Challenges and opportunities

While the promise of SBI and OI is immense, the field faces several challenges. This starts with a lack of common nomenclature, aka ontology (Kagan et al., 2024). Reproducibility of synthetic biological models, the efficiency of AI algorithms in interpreting complex biological data, and the integration of these systems into existing research and clinical pipelines remain hurdles. Additionally, interfacing and controlling these biological systems from a bioengineering perspective is still largely uncharted.

The research presented in this Topic demonstrates that these challenges are not insurmountable. Innovative experimental frameworks and novel in-vitro models inspired by in-silico solutions provide a roadmap for overcoming these barriers. Engineering advancements in interfaces and hardware will further accelerate progress in this domain.

## Ethical and security considerations

As we stand on the brink of creating living, thinking systems that merge biological and digital realms, ethical considerations are paramount. The implications of SBI and OI extend beyond medicine and research to broader societal concerns, including privacy, consent, and the security of biological data. These topics require multidisciplinary dialogue and the establishment of robust ethical frameworks.

## Toward a new frontier in biocomputing

This Research Topic highlights the transformative potential of combining synthetic biology and artificial intelligence. It lays the groundwork for future exploration of themes such as unconventional computing, in-vitro modeling, and the integration of biology with digital engineering. As SBI and OI evolve, they will redefine our understanding of intelligence and push the boundaries of what is possible in technology and medicine.

As Topic Editors, we are inspired by the breadth of work showcased in this Research Topic and the passion of the researchers contributing to this burgeoning field. With continued collaboration, innovation, and ethical stewardship, synthetic biological intelligence and organoid intelligence will undoubtedly revolutionize our approach to understanding and solving complex biological challenges.

## Summaries of the five articles

1. *Brain organoids and organoid intelligence from ethical, legal, and social points of view*? (Hartung et al.): This review examines the ethical, legal, and social implications of using human brain organoids for research and Organoid Intelligence (OI). While brain organoids hold potential for modeling neurodevelopment and neurological diseases, they raise questions about consciousness, moral status, and governance. The paper highlights the need for interdisciplinary frameworks to balance scientific progress with public and ethical concerns. It calls for proactive governance and stakeholder engagement to guide responsible development in this promising field.
2. *Versatile Micro-Electrode Array to Monitor Human iPSC-Derived 3D Neural Tissues at Air-Liquid Interface*? (Stoppini et al.): This study introduces a novel Micro-Electrode Array (MEA) technology designed for 3D neural tissues derived from human-induced pluripotent stem cells (hiPSC). It addresses the limitations of traditional electrophysiological assessments by utilizing a flexible, porous MEA integrated into an air-liquid interface (ALI) culture system. The innovation enables long-term survival and monitoring of neural activity in engineered tissues, advancing their application in disease modeling and neurotoxicology.
3. *Assembloid Learning: Opportunities and Challenges for Personalized Approaches to Brain Functioning in Health and Disease*? (Mencattini et al.): This opinion article explores the potential of assembloids—integrated 3D structures of multiple organoids—for personalized neuroscience research. It discusses their use in modeling complex brain functions such as learning and memory, as well as their application in studying neurodevelopmental and neurodegenerative disorders. The authors also address challenges like methodological limitations and ethical concerns, advocating for innovative tools and strategies to enhance organoid-based approaches.
4. *Organoid Intelligence for Developmental Neurotoxicity Testing*? (Alam El Din et al.): This perspective highlights the use of OI in enhancing developmental neurotoxicity (DNT) testing by incorporating neuroplasticity mechanisms. Brain organoids, coupled with AI and machine learning, offer a human-relevant platform for assessing the impact of chemicals on brain development. The article underscores the integration of these tools into the DNT testing paradigm to bridge gaps in traditional assays, aiming to improve predictions of neurotoxic effects.
5. *Open and Remotely Accessible Neuroplatform for Research in Wetware Computing*? (Jordan et al.): This technology and code paper describes an open-access Neuroplatform designed for global research in wetware computing. It enables remote experiments on living neural networks, offering tools for long-term electrophysiological monitoring and stimulation. The platform supports advanced biocomputing research, integrating brain organoids with deep learning and reinforcement learning algorithms. The infrastructure provides an innovative approach for collaborative experiments, pushing the boundaries of biological neural networks and wetware computing.

We thank the authors for their visionary contributions and invite the scientific community to continue this exciting journey into the intersection of the biological and digital. Together, we will shape the future of intelligence.
